# Tibial Biomechanics and Adaptive Response to Mechanical Stimuli in the Green Iguana

**DOI:** 10.1093/iob/obaf036

**Published:** 2025-10-22

**Authors:** T B Arlowe, W Sawatwong, R Fu, H Yang, D Little, T B Lescun, M L Figueiredo, R P Main

**Affiliations:** Department of Basic Medical Sciences, College of Veterinary Medicine, Purdue University, 625 Harrison St, West Lafayette, IN 47907, USA; Weldon School of Biomedical Engineering, Purdue University, 206 S. Martin Jischke Dr, West Lafayette, IN 47907, USA; Department of Biomedical Engineering, College of Chemistry and Life Science, Beijing University of Technology, 100 Pingleyuan, Chaoyang District, Beijing 100124, China; Department of Biomedical Engineering, College of Chemistry and Life Science, Beijing University of Technology, 100 Pingleyuan, Chaoyang District, Beijing 100124, China; Department of Basic Medical Sciences, College of Veterinary Medicine, Purdue University, 625 Harrison St, West Lafayette, IN 47907, USA; Weldon School of Biomedical Engineering, Purdue University, 206 S. Martin Jischke Dr, West Lafayette, IN 47907, USA; Department of Veterinary Clinical Sciences, College of Veterinary Medicine, Purdue University, 625 Harrison St, West Lafayette, IN 47907, USA; Department of Basic Medical Sciences, College of Veterinary Medicine, Purdue University, 625 Harrison St, West Lafayette, IN 47907, USA; Department of Basic Medical Sciences, College of Veterinary Medicine, Purdue University, 625 Harrison St, West Lafayette, IN 47907, USA; Weldon School of Biomedical Engineering, Purdue University, 206 S. Martin Jischke Dr, West Lafayette, IN 47907, USA

## Abstract

Mechanical loading models are used to study adaptive skeletal mechanobiology mechanisms. However, most studies have used mammal models, leaving a knowledge gap regarding how these mechanisms differ among vertebrate groups. To address this gap, we evaluated the *in vivo* bone strain environment of the left tibia in green iguanas during locomotion, axial compressive loading, and with finite element analysis. Our study involved examining male green iguanas (*n* = 7) over a range of speeds (0.45–1.34 m/s) and axial load magnitudes (–25 to –100 N) to determine peak strains. Bone strains were measured using single-element strain gauges and rosette strain gauges, surgically attached to the tibial anterior, posterior, and medial surfaces. At a speed of 1.34 m/s, peak strains ± standard deviation observed were 645 ± 699 µε, –448 ± 464 µε, and 206 ± 168 µε at the anterior, posterior, and medial surfaces, respectively. Peak principal tensile and compressive strains on the medial surface were 199 ± 113 µε and –153 ± 98 µε at 1.34 m/s. During –100 N compressive loading, peak strains were 403 ± 277 µε, –506 ± 460 µε, and –52 ± 177 µε at the anterior, posterior, and medial surfaces, respectively. Our finite element model demonstrated a close correlation with experimentally measured strain values at the gauge sites (slope = 1.07, *R* = 0.8381). Using these foundational *in vivo* strain results and a daily strain stimulus formula, our objective was to develop a novel noninvasive axial compressive tibial loading model to induce a cortical bone adaptive response in the green iguana tibia (*n* = 9). However, following 3 weeks of daily applied compressive loading, no significant difference was detected in critical bone parameters at 37 and 50% (midshaft) volume of interests from the proximal tibia (*P* < 0.05). While this study did not yield significant differences in critical bone parameters following the application of daily compressive loading, it provided new knowledge regarding the bone strain environment and the potential for inducing adaptive responses in the green iguana tibia. Further research may refine our understanding of skeletal mechanobiology mechanisms across vertebrate groups and develop more effective loading models for studying bone adaptation. Overall, the findings of this study, although limited, contribute to the broader field of musculoskeletal mechanobiology, giving insights that may inform bone health and adaptation in diverse species.

## Introduction

Foundational insights into the relationship between daily mechanical stimuli and a bone’s functional morphology (Rubin and Lanyon 1985; Wolff et al. 1986; Rubin and Lanyon 1987; Beaupre and Carter 1990a, 1990b; Frost 1997) have led to a concentrated and ongoing effort to understand how mechanical forces are transmitted within bone and the sensitivity of the bone’s adaptive response to mechanical stimuli. This effort has been critical in advancing our understanding of (1) biomechanical loading patterns during critical activities such as locomotion (Biewener et al. 1983; De Souza et al. 2005) and when bone is subjected to varying experimentally applied loads (Lanyon and Rubin 1984) and magnitudes (Sugiyama et al. 2012) and (2) in developing mechanical loading models to study the sensitivity of the *in vivo* physiological influence that mechanical load has on the (re)modeling of bone (Main et al. 2020). Understanding bone biomechanics provides a framework for comparison of the complexity of locomotor biomechanics in diverse vertebrate groups. The effect strain, the displacement of a bone surface in response to mechanical forces, reported as microstrain (µε, strain(ε), 10^–6^), has on these mechanobiology mechanisms is not fully established, and it is unknown if there is a differential (re)modeling response among vertebrates.

Terrestrial animals evolved different limb postures during locomotion, generally described as sprawling in ectotherms to upright or parasagittal in endotherms, with ectotherms changing posture as locomotor speed increases (Blob and Biewener 1999; Blob and Biewener 2001). This broad generalization of limb posture is not exact, as mammals such as monotremes can exhibit sprawling during locomotion (Fish et al. 2001; Brocklehurst et al. 2022). While previous studies have focused on mammals or birds, other studies have provided insights into the locomotor biomechanics of ectothermic species. Locomotor studies show that ectothermic vertebrate groups have higher safety factors and heavier skeletons relative to endothermic groups such as mammals and birds (Blob and Biewener 1999; Butcher et al. 2008; Sheffield and Blob 2011; Sheffield et al. 2011; Blob et al. 2014). This difference is attributed to variations in bone turnover rates and *in vivo* strain loading patterns between these groups (Blob and Biewener 1999; Blob et al. 2014). *In vivo* strain data demonstrate that the hindlimbs of green iguanas (*Iguana iguana)* and American alligators (*Alligator mississippiensis*) experience more torsional strain in the femur compared to birds and mammals (Blob and Biewener 1999). In the green iguana, the fibula is loaded primarily in compression and bears more mechanical load during locomotion than animals remaining upright during locomotion (Blob and Biewener 1999). One previous study examining the *in vivo* tibial bone strain during locomotion of green iguanas (Blob and Biewener 1999) reported results during both slow walks and high-speed sprints. Further characterizing both the *in vivo* bone strain and the hindlimb biomechanics of a reptile species such as the green iguana, across a linear increase in speed could enhance our understanding of biomechanical adaptation to upright locomotion in ectotherms.

*In vivo* mechanical bone loading models have contributed to our knowledge of skeletal mechanobiology mechanisms and musculoskeletal health. However, most have limitations, including the need for invasive osteotomies to targeted bones (Chamay and Tshantz 1971; Rubin and Lanyon 1987), observation of responses in cortical (Rubin and Lanyon 1987) or cancellous (Van der Meulen et al. 2006) bone alone, and reliance solely on mammalian or avian models (Main et al. 2020). One model, the murine axial tibial compressive loading model, is a noninvasive model that allows for the characterization of both cortical and cancellous tissue compartments and offers a high level of control over specific loading parameters such as strain magnitude, strain rate, rest periods, number of cycles per loading session, preloading magnitude, number of cycles per week, and control over the entire length of the loading study (Main et al. 2020). Additional key benefits of this model are that it allows animals to be returned to normal daily activities between applied loading sessions due to its noninvasive nature. Unlike direct loading to a specific periosteal surface, which has been shown to inhibit an endocortical response (Akhter et al. 1998), this model allows characterization of both endocortical and periosteal surfaces, and permits highly repeatable bending strains distributed in a physiological manner for the mouse tibia (Main et al. 2020). This is achieved by applying an external mechanical load through custom fixtures that are fitted to secure the knee and ankle joints of the animal’s hindlimb in varying positions (Main et al. 2020).

Currently, the axial tibial compression loading model is reported almost exclusively in mice (De Souza et al. 2005; Fritton et al. 2005; Ko et al. 2012; Toracasio et al. 2012; Main et al. 2020), but it has been applied also to chukar partridges (Verner 2017). This model is effective in producing controlled, repeatable loading protocols that can be applied across vertebrate groups to broaden the diversity of sample systems.

In the field of mechanobiology and musculoskeletal health, reptiles and nonmammalian species diverge from previous models, yet they remain relatively understudied. As a result, there is a knowledge gap regarding how these mechanobiology mechanisms might lead to differing responses to mechanical stimuli among vertebrate groups, particularly reptiles. Two key disadvantages of solely relying on mammalian or avian models are (1) it may limit our understanding of how these mechanisms are differentially regulated, and (2) it may impede our ability to recognize how physical adaptations impact skeletal structure and function across a comprehensive range of animal species. Broadening this knowledge to include more species can provide a critical test of the effectiveness of different approaches in improving musculoskeletal health.

**Fig. 1:**
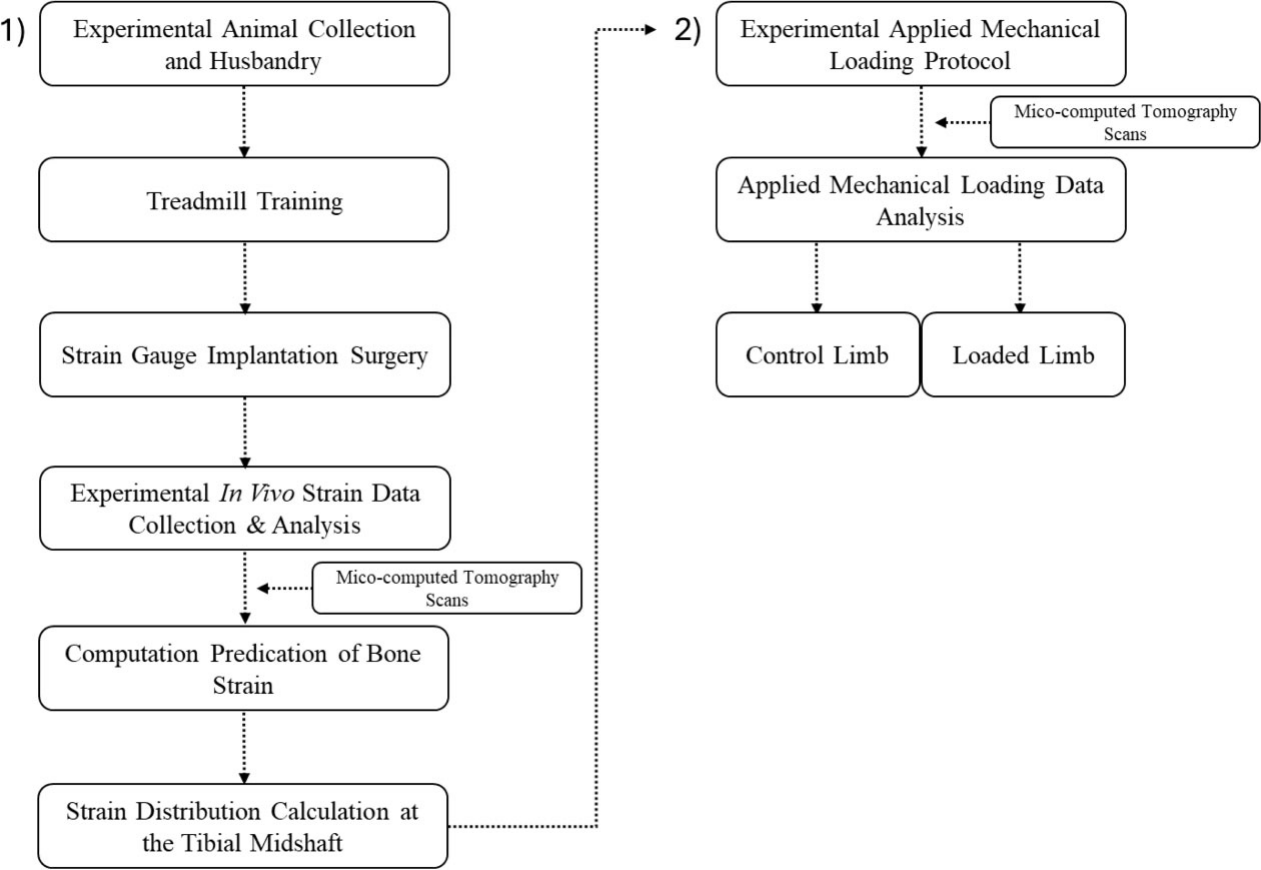
Flowchart summarizing the overall design of this study. (1) The steps in sequential order for characterization of the *in vivo* tibial strain treadmill locomotion, axial compressive loading with strain gauge measures, and finite element analysis. (2) The sequence of steps taken to analyze the morphological response of tibial cortical bone following a multiweek, noninvasive, applied axial compressive mechanical loading protocol. *Note*: The characterization of the *in vivo* tibial strain environment in (1) was utilized to determine the applied mechanical loading protocol in (2).

To bridge this gap, we aimed to develop a novel noninvasive, axial compressive reptilian tibial loading model to supplement existing models, using the green iguana as a representative species. The objective of this study was to characterize the tissue-level response of the reptile tibia to similar functional strain levels applied to mammals and birds in prior studies. We evaluated the *in vivo* bone strain environment at the cortical midshaft of the green iguana tibia during treadmill locomotion, noninvasive axial compressive loading, and finally with finite element analysis (FEA). Together, we utilized this data to characterize the morphological adaptive response in cortical bone following a daily applied noninvasive, axial-compressive loading protocol. A flowchart summarizing the study’s sequential design is shown in Fig. 1, including first characterizing the *in vivo* strain environment and subsequentially the morphological cortical response of the green iguana tibia.

## Material and methods

### Experimental animal collection and husbandry

All procedures described were approved by the Purdue University IACUC (Protocol # 2105002148A001). Male green iguanas were collected in Boca Raton, Florida, specifically on the Florida Atlantic University (FAU) campus and the surrounding Boca Raton area, captured by trained individuals using a constructed catch pole. Once restrained, iguanas were physically examined to determine health, sex, and size. The target size of iguanas was similar to those described previously (Blob and Biewener 1999). Physical examinations to determine health included checking for missing limbs and digits, as well as any external wounds. Limb palpations were assessed for skeletal abnormalities, and whole-body movement was also evaluated during capture. The sex of the iguanas was determined by observing the presence of external femoral pores. If the iguana was determined to be healthy and male, the measurements collected included mass (kg), snout-vent length (SVL, mm), and left tibial length (TL, mm) from the femorotibial joint to the tibiotarsal joint (Tables S1 and S2). Green iguanas were placed in soft, white cotton cases and housed in individual pet carriers (Petmate Two-Door, Top and Front-Loading Pet Carrier, 24″ L), and were transported back to the Purdue University College of Veterinary Medicine Animal Holding Facility in West Lafayette, IN, USA. Captured iguanas remained in their carriers at all times with the temperature maintained between 21 and 27°C and transported to our facilities within 72 h. All relevant approvals and permits for capture and transport were obtained from the Florida Fish and Wildlife Conservation Commission and the states along the transportation route.

Upon arrival, all iguanas were treated with a topical spray (Miracle Care Reptile Mite Spray) for surface parasites and were also treated twice with Profender Topical Solution (1 mL/kg; 21.4 mg/mL emodepside/85.8 mg/mL praziquantel, Elanco US Inc., Greenfield, IN, USA) for internal parasites. They were individually housed, provided with enrichment tree branches (sourced from local available habitat) and shelving for daily activities, a UVB light source (UVB100, 18 W, 24″; 12 h on, 12 h off), a heat lamp (75 W) for daily basking, consistent temperatures (29–35°C), adequate daily water, and a diet of fresh chopped greens, vegetables, and fruit.

Iguanas were monitored daily for any signs of distress or illness, such as weight loss, abnormal fecal appearance, or abnormal behaviors, and their body weight was measured weekly. All iguanas were given at least 1 week to acclimate to their new environment before any handling for data collection.

### Experimental *in vivo* strain

#### Treadmill training

Iguanas were trained to run on a customized treadmill (Woodway Inc., Waukesha, WI, USA) with a fitted plywood and plexiglass box installed to prevent them from running off the treadmill. Treadmill training consisted of individual training sessions 3×/week for at least 4 weeks until individual iguanas ran consistently for multiple trials at speeds ranging from a walk to a sprint.

The plexiglass box allowed tracking of Qualysis Track Manager (QTM) markers and filming black and white video during data collection to determine the stance phase of the iguana gait and observe consistent footfalls, similar to previous studies (Reilly and Elias 1998).

Iguanas were encouraged to run by a researcher standing at the back of the treadmill and tapping the treadmill behind them to direct them to run toward the dark, shaded area located at the front. Training trials at each speed lasted until the iguana was tired, indicated by falling toward the back of the treadmill, or until 10 s of consistent running, whichever was earlier. For all speeds, no trial lasted longer than 10 s to account for the length of data collection and the stamina of iguanas. Each trial speed and the iguana’s corresponding running behavior were recorded. A training trial was considered successful when the iguana ran consistently in the middle of the treadmill for at least five steps. A top speed was determined for each iguana as the highest speed at which the iguana could complete ≥3 successful trials. Once the iguana ran ≥3 successful trials at each speed for three consecutive training sessions, it was considered ready for data collection.

#### Strain gauge implementation

Aseptic surgery was conducted to attach strain gauges on the left tibia of each iguana (*n* = 7, Mass: 0.88 ± 0.19 kg, SVL: 278.57 ± 38.73 mm, TL: 54.29 ± 4.89 mm; raw measurements (Table S1). All surgical procedures were based on previous detailed techniques (Blob and Biewener 1999; Main and Biewener 2004; Main and Biewener 2007). General anesthesia was induced via an intramuscular injection (IM) of 10–20 mg/kg alfaxalone, evenly divided between both forelimbs (Bertelsen and Sauer 2011). A local subcutaneous injection (SQ) of 5 mg/kg lidocaine was performed at the incision sites. A 14 G × 120 mm IV catheter (Delta Med. S.p.A., Viadana, MD, Italy) was inserted for endotracheal intubation, with anesthesia maintained with 1.5–5.0% isoflurane at a 0.4 L/min O_2_ flow rate. To monitor breathing and heart rate, an ultrasonic Doppler flow monitor was taped to the ventral surface of the animal’s chest, and the anesthesia depth was adjusted as necessary. Throughout the surgery, a recirculating heating pad set to 90°F was placed underneath the surgical drapes to maintain the body temperature of the iguana.

The left tibial midshaft was measured and marked from the proximal and distal ends of the tibia, and a longitudinal incision was made in the medial surface of the crus at the level of the midshaft. An additional incision was made on the dorsal surface of the pelvis, and a subcutaneous tunnel was created from the hip to the tibia. The musculature overlying the tibial midshaft was retracted to expose the anterior, posterior, and medial surfaces for strain gauge implantation. The bone surface was prepared for attachment by gently scraping with a periosteal elevator to remove the periosteum, cleaned with 2-butanone and left to dry. Strain gauges were passed subcutaneously from the pelvis incision to the tibial midshaft. The underside of each gauge was cleaned with 2-butanone and allowed to dry before being attached to the bone using a self-catalyzing cyanoacrylate adhesive (DURO Superglue, Loctite, Westlake, OH, USA). Single-element strain gauges (FLKB-1-11, Tokyo Measuring Instruments Laboratory Co., Ltd., Shinagawa-ku, Tokyo) were attached to the anterior and posterior surface. Following examination of the medial surface width, either a single-element (FLKB-1-11) or rosette (FRAB-1-11, Tokyo Measuring Instruments Laboratory Co., Ltd.) gauge was attached. The gauges were centered on each surface, and the single-element gauge or central gauge of the rosette was aligned with the longitudinal axis of the tibia within 5°. After the gauges bonded to bone, the previously retracted musculature was carefully placed back, and both incisions were sutured. The lead wires of the strain gauges exiting the pelvic incision were sutured to the skin at the dorsal surface of the tail base to minimize tension during locomotion and ensure the bond security of gauge and bone. Gauze was placed underneath a presoldered breakout plug (4-103240-0, Digi-Key, Thief Rivers Falls, MN, USA), attached to the gauge wires, to prevent friction with the iguana’s skin. Both the incision and plug were covered with elastic bandage tape. Immediately following surgery, a single SQ injection of 0.2 mg/kg meloxicam was injected into one of the forelimbs of each iguana to manage postoperative pain, swelling, and inflammation.

During recovery, each iguana was placed in their individual pet carrier while the carrier was kept on the same heating pad from surgery and monitored 1–2 times/h for the next 5 h. Once the iguanas had their eyes opened, were ambulatory and indicated no sign of lameness, they were returned to their individual housing unit.

#### Experimental *in vivo* strain data collection

*In vivo* bone strains were collected during treadmill locomotion and noninvasive, axial-compressive loading 24 h after surgery. The following morning after surgery, another single SQ injection of 0.2 mg/kg meloxicam was administered before data collection, and each iguana was assessed again for any signs of lameness. Prior to treadmill locomotion, the presoldered breakout plug was connected to a shielded coaxial cable (NMuF 6/30-404655, Cooner Wire, Chatsworth, CA, USA), which was connected to a bridge amplifier (2120B, Micro-measurement, 2110B Power Supply Model, Vishay Precision Group, City of Industry, CA, USA) and A/D converter (AD Instruments, Dunedin, NZ, USA). This allowed for strain gauge signals to be sampled at 2000 Hz. The raw output voltage from the strain gauges was arithmetically converted to µε using LabChart7 software (AD Instruments, Dunedin, NZ, USA) and zero-balanced simultaneously while the iguanas were stationary. Strain data was collected simultaneously while each iguana ran for 10-s intervals over a range of speeds from a walk to top speed (0.45–1.34 m/s). Hindlimb posture was confirmed visually during data collection and video recording, with a sprawling posture observed during slow walking and an upright posture during fast sprinting. As a novel study during incremental speed increases, limb posture was assumed to gradually transition from a sprawling posture to a more upright posture.

**Fig. 2:**
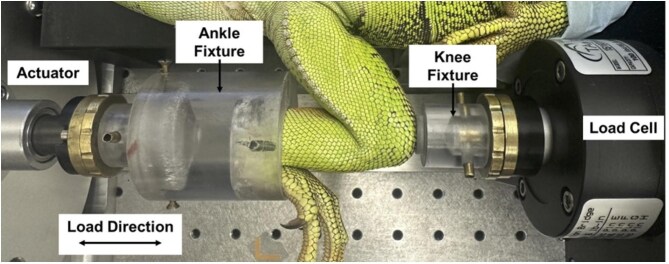
Configuration of both the left ankle and knee joint of the iguana in the custom mechanical loading fixtures viewed from the ventral aspect. The cyclic load was applied from the actuator through the ankle joint as indicated. The iguana was placed in a supine position with a horizontally oriented tibia.

Immediately after treadmill locomotion, iguanas were anesthetized and intubated using the same procedures as those used during strain gauge surgeries. The left knee and ankle of each iguana were positioned into custom mechanical loading fixtures for axial compressive loading (Fig. 2). *In vivo* bone strains were collected while the limb was loaded cyclically at 4 Hz with a triangular waveform, starting with a –10 N preload and increasing maximum load magnitudes (–25, –50, –75, and –100 N). The load was applied at the ankle from the actuator and transferred through the tibia and knee to the load cell (Fig. 2).

*In vivo* strain data was collected at 2000 Hz, using the same presoldered breakout plug, shielded coaxial cable, bridge amplifier, and A/D converter used during locomotion. Loading data was simultaneously collected at 2000 Hz from the mechanical loading instrument (200 N Bose ElectroForce TestBench System, TA Instruments, New Castle, DE, USA).

Following completion of axial compressive loading, anesthesia was maintained, and iguanas were euthanized via an intracardiac injection of 500 mg/kg sodium pentobarbital (Knutson et al. 2022; Wong et al. 2024) and immediately frozen for future bone imaging and FEA.

#### Custom mechanical applied loading fixtures

Custom mechanical loading fixtures were constructed for both the ankle and knee. These fixtures were implemented in an axial compressive loading model (Yang et al. 2014; Main et al. 2020) on the left tibia of green iguanas. The ankle and knee fixtures are shown in Fig. 2 and were made from clear impact-resistant polycarbonate rods in 2.0″ and 1.0″ diameters, respectively.

#### Experimental *in vivo* strain data analysis

To analyze treadmill locomotion raw strain, four consecutive, steady strides were selected from one to two trials at every speed for every iguana for a total of *n* = 4–8 steps to be analyzed. Within a custom MATLAB program, a fourth-order Butterworth filter was applied to raw strain data and zeroed by averaging the strain during the entire swing phases for all the selected strides. Peak longitudinal strains were determined from the single-element gauges and the rosette central gauge for all surfaces and their occurrence during stance. Peak principal tensile and peak principal compressive strains and their orientation relative to the longitudinal axis of the tibia were calculated for the medial surface using standard equations (Biewener 1992).

For analysis of the axial compressive loading, a separate MATLAB program was created to calculate the peak longitudinal strains at each applied load magnitude. Raw strain was zeroed by averaging the strain level immediately prior to the –10 N preload and after the strain gauges were balanced. Two trials were selected for each load magnitude, and four consecutive load cycles were selected from each trial for a total of *n* = 8 cycles to be analyzed at each bone surface. To assess this relationship, we analyzed a linear regression and Spearman correlation (*R*) between axial compression load magnitude and mean peak longitudinal strains for each bone surface.

Our analysis continued with utilizing the Froude Number, a dimensionless speed parameter used to compare subjects of varying morphologies (Biewener 2003). This parameter was key in facilitating the comparison of *in vivo* strain within our test group. Each iguana’s locomotion at five speeds was categorized into Froude Class 1 (lowest speed) through Froude Class 5 (highest speed). To calculate the total leg length (mm) used in our Froude calculations for each iguana, we measured the summation of hip to knee length (mm), knee to ankle (mm), and ankle to fourth or fifth toe (mm). These anatomical landmarks have been described previously (Reilly and Elias 1998). Froude classes with corresponding Froude numbers (mean ± one standard deviation) are as follows: Froude Class 1 (0.114 ± 0.005), Froude Class 2 (0.247 ± 0.019), Froude Class 3 (0.431 ± 0.033), Froude Class 4 (0.689 ± 0.05), and Froude Class 5 (0.986 ± 0.072). A one-way analysis of variance (ANOVA) or Kruskal–Wallis statistical analysis was performed based on the *F* test, skewness, and kurtosis to assess differences in longitudinal strain across varying Froude classes. Normality assumptions were evaluated using the Shapiro–Wilk normality test of the residuals and the Levene’s test for homogeneity of variance for the parametric ANOVA.

#### Computational prediction of bone strain

The strain-gauged left tibia of each iguana was scanned using a PerkinElmer Quantum GX micro-computed tomography (µCT) (Quantum GX, PerkinElmer, Waltham, MA, USA). Limbs were scanned with the gauges still bonded to the bone to determine exact gauge location during finite element (FE) modeling. The intact knee and ankle joints were placed into the same custom mechanical loading fixtures and positioned similar to their orientation during *in vivo* axial compressive mechanical loading. This setup allowed for joint contacts and boundary conditions to be determined during FE modeling.

**Fig. 3:**
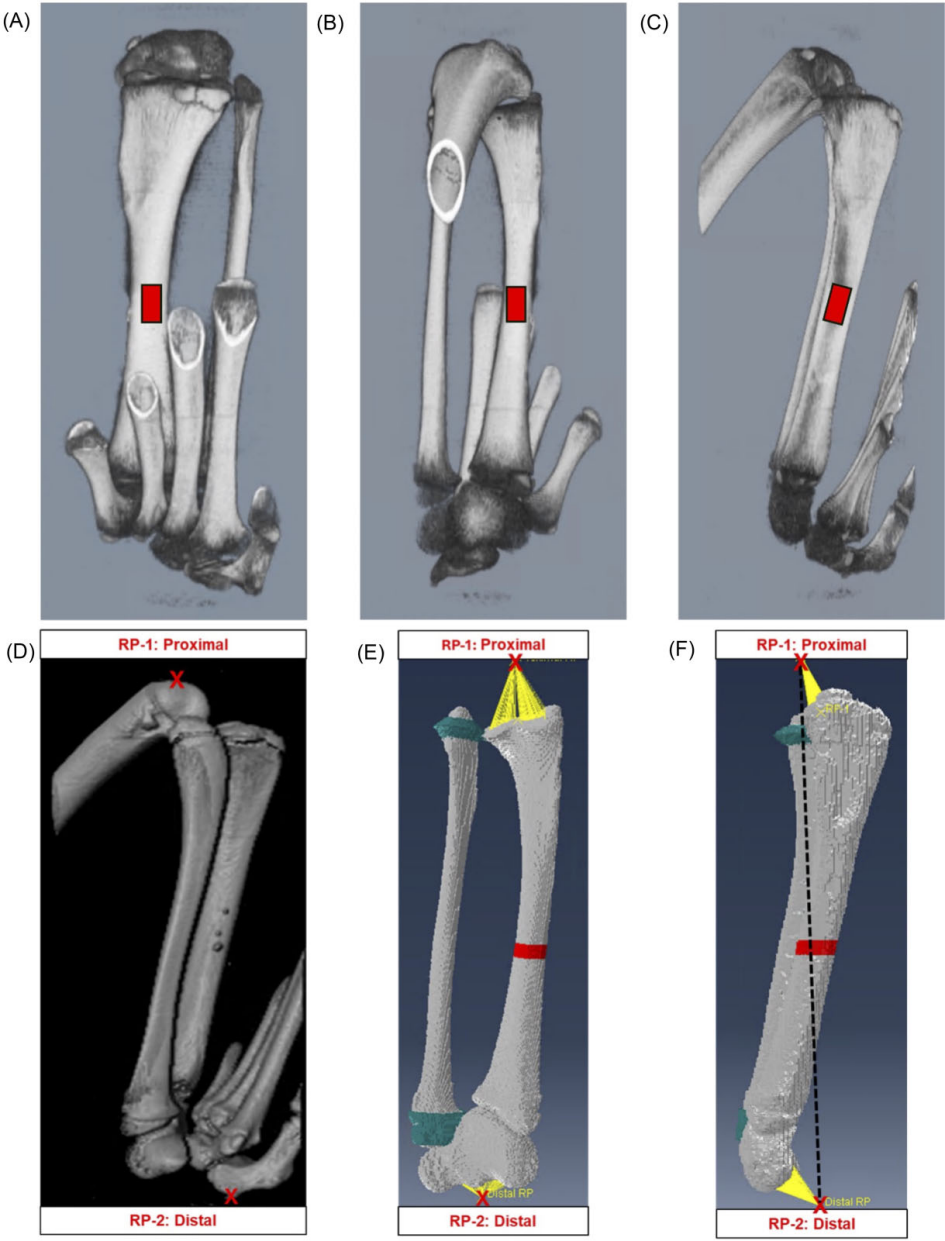
For images **A, B**, and **C**, whole limb µCT images stitched together using XamFlow software for finite element analysis (FEA). (**A**) anterior surface, (**B**) posterior surface, (**C**) medial surface. The mid-diaphyseal squares indicate each strain gauge location bonded for each bone surface (three strain gauges per iguana, one strain gauge per bone surface). For images D, E, and F, FE model determination of tibial boundary conditions and direction of applied axial compressive mechanical loading. Reference points 1 (RP-1) and 2 (RP-2) are indicated by an X, and RP-1 is proximal while RP-2 is distal relative to the tibia in all images. (**D**) Stitched microcomputed tomography (µCT) of the iguana tibia in the lateral view, a threshold value was determined to separate bone from soft tissue and background. (**E**) FEA model of the iguana tibia and fibula in the cranial view using Abaqus software; the midshaft volume of interest (VOI) is indicated in red. (**F**) Medial view of the iguana tibia relative to the reference points, and the direction of applied compressive mechanical load is indicated by the black dotted line connecting both reference points; the midshaft VOI is indicated mid-diaphysis.

Initial scans were taken with an isotropic voxel resolution size of 144 µm (90 kV, 88 µA, 14-min scan time, high resolution, Cu 0.06 + Al 0.5 X-ray filter, no frame averaging) of the knee, midshaft tibia, and ankle for a total of three scans per iguana. Initial scans were subreconstructed at a 60 µm resolution using Quantum CT software. Whole limb images were stitched together using XamFlow software, as shown in Fig. 3A–C. PerkinElmer-specific calibrations were performed using hydroxyapatite bone phantoms, which converted the microcomputed tomography (µCT) attenuation values to bone mineral density (BMD) (mg/cm^3^).

Iguana-specific FE models (*n* = 6) were developed for instrumented bones using the µCT image stacks. For each image stack, a threshold value was determined to separate bone from the background pixels using MIMICS (version 20.0, Materialise, Leuven, Belgium). Similar to the method used in our previous FE modeling studies of the mouse tibia (Yang et al. 2014; Yang et al. 2017; Yang et al. 2019; Yang et al. 2021), cartilage was used to fill the “gap” between the tibia and fibula. The “gap” between the tibia and fibula was observed in µCT scans because the two bones are capped with cartilage, which is radiolucent (Fig. 3E). A three-dimensional FE mesh (C3D4 tetrahedral element) model was created in MIMICS using the segmented tibial µCT stacks. The mesh provided a smooth bone surface favorable for surface strain calculation, especially in the region of the strain gauge. The material properties of bone were assumed to be linearly elastic, isotropic, and homogeneous. Young’s modulus (E) was set at 15 GPa and Poisson’s ratio (ʋ) was 0.3 for both tibia and fibula based on measured average elastic modulus of cortical bone (Yang et al. 2014). For the assigned cartilage between the tibia and fibula, the material properties were *E* = 100 MPa and ʋ = 0.3. Previous studies have shown that the influence of soft tissues, ligaments and cartilage on the calculated strains in FE models can be neglected (Yang et al. 2014). To determine boundary conditions, the proximal reference point (reference point 1: RP-1) was identified from contact on the anterior surface of the distal femur with the knee fixture, while the distal reference point (reference point 2: RP-2) was identified by contact of the central axis of the ankle fixture with the plantar surface of the tarsus (Fig. 3D–F). The longitudinal axis was defined as the line connecting between these two points. A concentrated compressive load of –100 N, representing the peak *in vivo* compressive load, was applied through the distal reference point. Linear elastic FEA was performed (Abaqus 6.14, Simulia) to simulate the tibial axial compressive loading configuration. Each model was validated against corresponding experimental strain results obtained at the strain gauge location.

After validation, the models were used to determine maximum and minimum principal tensile and compressive strains at the cortical midshaft (50% bone length) using 2.5% of bone length (∼1.5 mm) as the volume of interest (VOI) during loading (Fig. 3E–F). The value at the 95th percentile was used as the cutoff for the range of strain values induced during loading at the midshaft VOI.

#### Strain distribution calculation at the tibial midshaft

The direction of the neutral axis at the tibial midshaft for *in vivo* strain during experimental axial compressive loading was determined using a previously described method (Patel et al. 2014). This study involved four iguanas (*n* = 4), as these were the iguanas that had all three gauges attached during peak load (−100 N). Determination of the neutral axis at the tibial midshaft allows us to compare the bending orientation observed during *in vivo* compressive mechanical loading and our iguana-specific FE models.

#### Experimentally applied mechanical loading protocol

In order to assess the bone response to an applied mechanical axial loading protocol, a separate group of iguanas was used. Each iguana (*n* = 9, Mass: 0.97 ± 0.19 kg, SVL: 283.33 ± 21.77 mm, TL: 56.67 ± 3.65 mm; raw measurements) (Table S2) had their left limbs placed in the custom loading fixtures and were subjected to a noninvasive externally applied axial compressive load 5 days a week (Monday through Friday) for 3 weeks, with loading bouts occurring approximately 24 h apart. General anesthesia was induced by IM injection of 10–20 mg/kg alfaxalone, evenly divided between both forelimbs of each iguana. Anesthesia was maintained with 1.5% isoflurane at a 0.4 L/min O_2_ flow rate via anesthesia mask and adjusted as necessary. The left knee and ankle of each iguana were firmly secured into the same custom fixtures used for *in vivo* strain collection during axial compressive loading (Fig. 2). Once the left limb was secured, a –10 N preload was applied, followed by maximum cyclic loading of –75 N for 300 cycles with a triangular waveform at 4Hz. Each limb was closely examined during and after each loading bout to assess for any tissue or bone damage. On Day 22, 3 days after the last loading bout, each iguana was euthanized following the same protocol used after *in vivo* strain data collection. The iguanas were immediately frozen for subsequent tissue removal and analysis.

Previous studies have described the relationship between bone maintenance and strain, demonstrating that there is a minimum effective strain present on the bone to induce an adaptive response (Beaupré et al. 1990b; Turner et al. 1994; Frost 1997; De Souza et al. 2005;Yang et al. 2021). In previous murine tibial models, the diaphyseal cortical bone applied strain threshold was measured to be ∼+1,050 µε (Turner et al. 1994), while locomotor peak strains at the tibial midshaft were measured to range from ∼+400 to ∼–600 µε (De Souza et al. 2005). This indicates that the optimal load magnitude to elicit a bone adaptative response should induce peak strain ∼2.5× an animal’s daily locomotor peak strains.

To establish an experimentally applied mechanical loading protocol to apply similar functional strain levels to green iguanas, we utilized the *in vivo* and FEA results in this study and reported *in vivo* results from mechanical compressive loading in birds (Verner 2017) and hindlimb locomotor strain in other reptile species (Blob and Biewener 1999; Butcher et al. 2008) using the intracortical daily strain stimulus (Chen et al. 2010), resulting in 300 load cycles per day, with further details described in supplementary material (S3). A triangular waveform (ramp/unload timeframe: 0.15 s; load application frequency: 4 Hz) was applied to compare results to murine and avian models (Verner 2017; Main et al. 2020).

#### Applied mechanical loading data analysis

Following euthanasia, both the loaded (left) and nonloaded control (right) limbs of each iguana were dissected. Subsequently, the limbs were scanned using a PerkinElmer Quantum GX µCT for both initial (144 µm isotropic voxel resolution, 90 kV, 88 µA, 14-min scan time, high resolution, Cu 0.06 + Al 0.5 X-Ray Filter, no frame averaging) and subreconstruction (60 µm resolution) scans. Also described earlier, the knee and ankle joints were placed into the same custom mechanical loading fixtures and positioned similar to their orientation during axial compressive mechanical loading. As a novel loading protocol, scans of the knee and ankle joints were completed to ensure there was no structural damage to bones within those joints in addition to the tibia and fibula.

Immediately following the completion of scanning, tibiae were dissected to remove all tissue from the bone, fixed for ≈36 h in 10% neutral buffered formalin, and then stored in 70% ethanol at –4°C for future bone histomorphometry analysis. During dissection, no damage was macroscopically observed to the surrounding tissues following mechanical loading.

Whole limb images were stitched together using the Pairwise Stitching Plugin in BoneJ (Linear Blending, check peaks = 50) (Preibisch et al. 2009). The stitched images were prepared for bone morphometry analyses in Analysis 14.0. For each µCT image stack, the limbs were placed in the correct anatomical position in the transform tool, and a threshold value was determined to separate bone from the background pixels. Subsequently, the tibia was segmented from all other hindlimb bones using the object extractor tool. Following segmentation of the whole tibia, specific volumes of interest (VOIs) were segmented for bone analysis. Segmented VOIs in the Analyze 14.0 Bone Micro Architecture Add-on were applied to calculate specific parameters in cortical bone.

To measure the tissue-level cortical bone response, BMD, bone volume, total cross-sectional area, bone area, medullary area, bone area fraction, bone thickness, periosteal perimeter, endocortical perimeter, and maximum (*I*_max_) and minimum (*I*_min_) moments of inertia values were obtained (Bouxsein et al. 2010; Main et al. 2010; Sugiyama et al. 2010; Silva et al. 2012; Weatherholt et al. 2013). For bone morphometry analyses, bone volumes representing 2.5% of bone length at 37%, and 50% (midshaft) bone length relative to the proximal end of each tibia were selected. The VOI at 37% was selected because this is another site for strain gauge implementation and was previously described as the tibial site for an osteogenic response to axial compressive loading in mice (Sugiyama et al. 2010; Main et al. 2020).

To test the differences between parameters, paired Student’s *t*-tests or Wilcoxon signed-rank tests were performed between the loaded limb and the nonloaded control limb. Statistical significance was established at a *P*-value of less than 0.05 (*P* < 0.05). Normality was confirmed with a Shapiro–Wilk normality test of the within-pair differences.

### *In vivo* bone strain- results

#### Biomechanics of *in vivo* tibial strain distribution in iguanas during locomotion

**Fig. 4:**
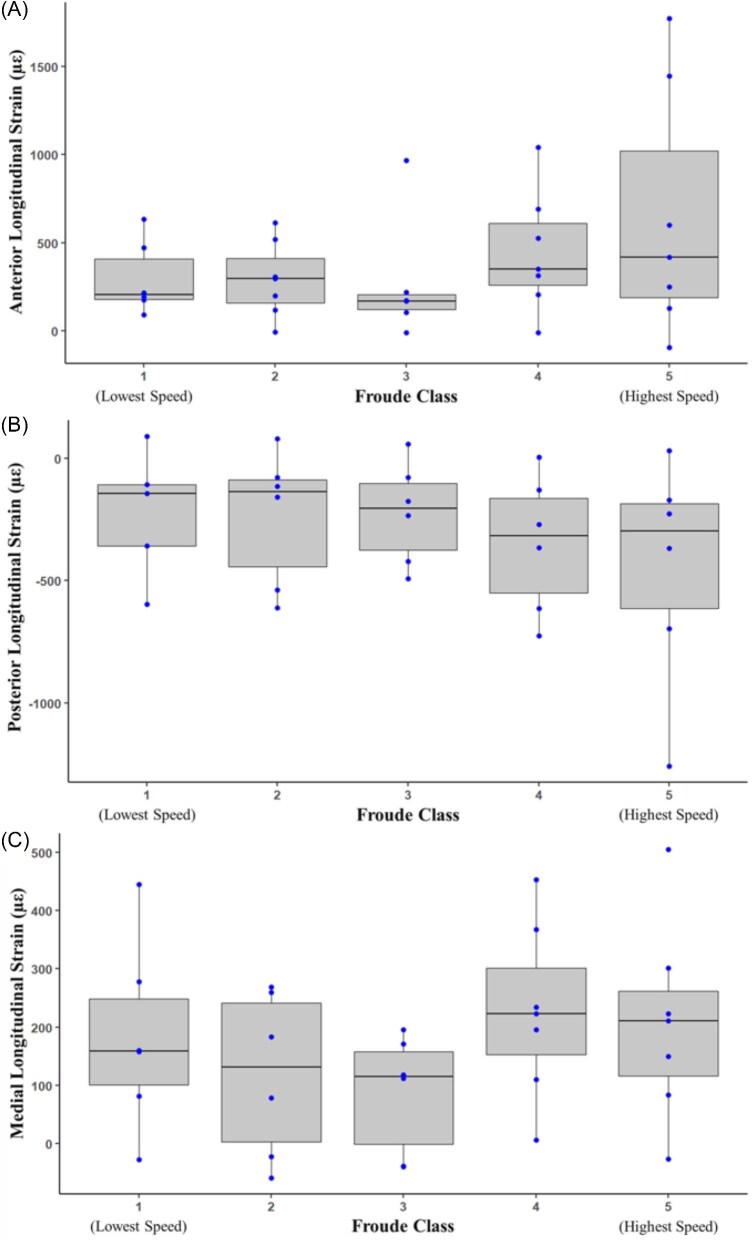
Box plots displaying *in vivo* longitudinal strain (µε) during treadmill locomotion with increasing Froude Class on the (**A**) anterior, (**B**) posterior, and (**C**) medial surfaces. Blue dots indicate mean strain values corresponding to individual iguanas during each Froude Class. The gray box plot displays the interquartile range from the 25th to 75th percentile, the black line represents the median value, and the whiskers represent values in the lower and upper range resulting from all trials analyzed during each speed. Any values outside the whiskers are indicative of extreme values. *Whiskers are present for Froude Class 3 on the anterior surface (**A**) but are very small and covered by blue scatter points.

*In vivo* tibial strains were measured from male green iguanas (*n* = 7) over a range of treadmill speeds (0.45–1.34 m/s). Peak strains observed at 1.34 m/s on the anterior, posterior, and medial surfaces, respectively, were 645 ± 699 µε, –448 ± 464 µε, and 206 ± 168 µε and occurred at 58 ± 10%, 52 ± 8%, and 39 ± 13% of stance, respectively (Table 1). Longitudinal peak strains on the anterior and medial surfaces were tensile, while the longitudinal peak strains on the posterior surface were compressive (Table 1, Fig. 4), indicating an anterior-medial to posterior-lateral bending direction during stance. Mean longitudinal strain did not significantly change for posterior (one-way ANOVA: *F* = 0.6013, *df* = 4 and 24, *P* = 0.6653) and medial (one-way ANOVA: *F* = 1.0394, *df* = 4 and 27, *P* = 0.4052) surfaces with increasing speeds. Longitudinal strain did not increase with speed for the anterior surface (Kruskal–Wallis: *H* = 3.1793, *df* = 4, *P* = 0.5283).

**Table 1:** *In vivo* mean peak longitudinal strains (µε) for the anterior, posterior, and medial surfaces at all speeds (m/s) during treadmill locomotion and its occurrence during stance (%).

**Froude class**	**Speed (m/s)**	**Iguanas (*n*)**	**Anterior (µε)**	**Anterior stance (%)**
1	0.45	6	296 ± 209	54 ± 8
2	0.67	7	291 ± 217	56 ± 12
3	0.89	6	269 ± 351	51 ± 10
4	1.12	7	445 ± 345	51 ± 10
5	1.34	7	645 ± 699	58 ± 10
**Froude class**	**Speed (m/s)**	**Iguanas (*n*)**	**Posterior (µε)**	**Posterior stance (%)**
1	0.45	5	–224 ± 261	45 ± 7
2	0.67	6	–237 ± 274	52 ± 12
3	0.89	6	–224 ± 206	50 ± 7
4	1.12	6	–349 ± 280	56 ± 11
5	1.34	6	–448 ± 464	52 ± 8
**Froude class**	**Speed (m/s)**	**Iguanas (*n*)**	**Medial (µε)**	**Medial stance (%)**
1	0.45	6	183 ± 163	41 ± 14
2	0.67	6	118 ± 141	42 ± 11
3	0.89	6	87 ± 102	45 ± 8
4	1.12	7	227 ± 150	42 ± 10
5	1.34	7	206 ± 168	39 ± 13

Positive values indicate tensile strain, while negative values indicate compressive strain. Values represent mean strain ± one standard deviation.

On the medial surface, at the highest speed (1.34 m/s), the peak principal tensile strain (E1) was 199 ± 113 µε and oriented –3° ± 21 (Table 2), acting in a proximal to a distal direction. Similarly, at the same speed, the peak principal compression strain (E2) was –153 ± 98 µε and oriented –93° ± 6, acting in an anterior to posterior direction (Table 2).

**Table 2:** *In vivo* mean peak principal tension (E1), mean angle of peak tension relative to the medial longitudinal axis of the tibia (ϕ1), mean peak principal tension occurrence during stance (%), and mean longitudinal strain (µε) from the central rosette gauge. *In vivo* mean peak principal compression (E2), mean angle of peak compression relative to the medial longitudinal axis of the tibia (ϕ2), mean peak principal compression occurrence during stance (%), and mean longitudinal strain (µε) from the middle rosette gauge.

**Froude class (*n* = iguanas)**	**Peak tension (E1)**	**Tension angle (ϕ1)**	**E1 stance (%)**	**Longitudinal (µε)**
1 (2)	240 ± 142	10 ± 34	37 ± 7	180 ± 139
2 (2)	104 ± 79	12 ± 32	33 ± 8	10 ± 97
3 (3)	191 ± 73	19 ± 22	35 ± 3	82 ± 108
4 (3)	239 ± 92	5 ± 21	43 ± 4	189 ± 69
5 (3)	199 ± 113	–3 ± 21	40 ± 2	148 ± 63
**Froude class (*n* = iguanas)**	**Peak compression (E2)**	**Compression angle (ϕ2)**	**E2 stance (%)**	**Longitudinal (µε)**
1 (2)	–146 ± 109	–73 ± 23	43 ± 2	180 ± 139
2 (2)	–98 ± 2	–62 ± 24	47 ± 11	10 ± 97
3 (3)	–143 ± 68	–74 ± 12	47 ± 2	82 ± 108
4 (3)	–184 ± 96	–79 ± 10	60 ± 2	189 ± 69
5 (3)	–153 ± 98	–93 ± 6	50 ± 4	148 ± 63

Values represent mean ± one standard deviation.

#### *In vivo* biomechanical response of tibial surfaces to axial compressive loads

**Fig. 5:**
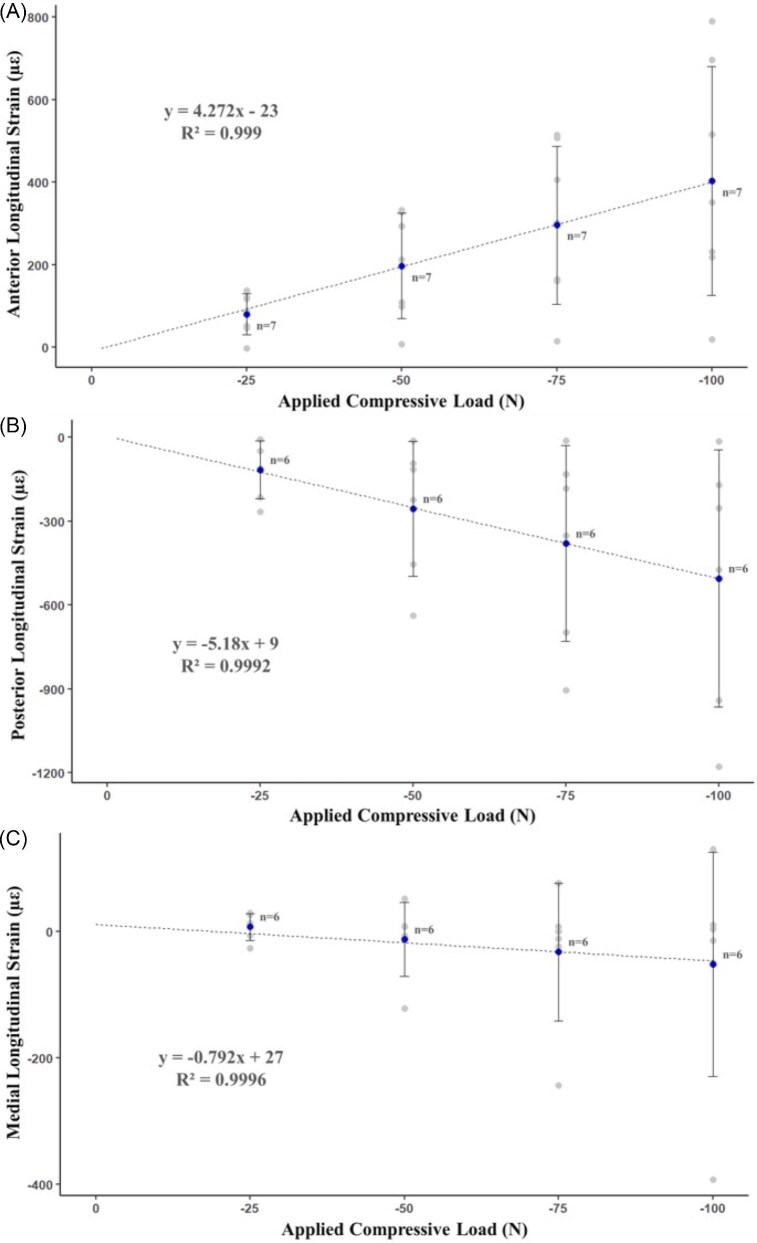
Scatterplot displaying *in vivo* mean peak longitudinal strains (µε) during applied axial compressive load (from –25 to –100 N) on the (**A**) anterior, (**B**) posterior, and (**C**) medial surfaces. A trendline and equation are displayed. The sample size during each load magnitude is indicated along with its corresponding value. Blue plot points represent mean peak strain during loading, and error bars represent ± one standard deviation. Gray plot points represent mean peak strain during load for each individual iguana. Positive values represent tensile strain, whereas negative values represent compressive strain. The dotted line represents trendline; the equation, along with the *R*-squared value, represents results from linear regression.

Longitudinal mean peak strains on the anterior, posterior, and medial surfaces increased with increased load magnitudes during experimentally applied axial compressive loading (Fig. 5, Table 3). The linear regression relationships for the anterior, posterior, and medial surfaces were *y* = 4.272*x* – 23 (*R*^2^ = 0.999), *y* = –5.18*x* + 9 (*R*^2^ = 0.999), and *y* = 0.792*x* + 27 (*R*^2^ = 0.999) (Fig. 5), respectively. On the anterior surface, the longitudinal strain was tensile, while longitudinal strains on the posterior surface were compressive (Fig. 5, Table 3), indicating bending in the anterior-posterior direction, similar to the bending pattern during locomotion. Longitudinal strain on the medial surface was in tension during low magnitudes of load and was in compression with increasing load magnitudes (Fig. 5, Table 3), indicating the medial gauge was near the neutral axis and the anterior-posterior bending increased in the medial-lateral direction with increasing load magnitudes. Mean longitudinal strain for each surface and its corresponding load magnitude is displayed in Table 3. *In vivo* longitudinal strain during peak axial compressive loading (–100 N) did not result in any of the surfaces reaching 2.5× the *in vivo* longitudinal strain observed during treadmill locomotion (Table 4).

**Table 3:** *In vivo* mean peak longitudinal strains (µε) were measured during applied axial compressive loads (–25 to –100 N) for the anterior, posterior, and medial surfaces.

Compressive load (*N*)	Anterior (µε)	Posterior (µε)	Medial (µε)
–25	80 ± 50	–116 ± 103	7 ± 21
–50	197 ± 128	–256 ± 242	–12 ± 59
–75	296 ± 191	–381 ± 250	–33 ± 109
–100	403 ± 277	–506 ± 460	–52 ± 177

The values represent mean peak strain ± one standard deviation. For the anterior surface, *n* = 7, and for the posterior and medial surfaces, *n* = 6 measurements were made.

**Table 4:** *In vivo* mean peak longitudinal strains (µε) during the highest Froude class of treadmill locomotion and peak applied axial compressive load (–100 N) for the anterior, posterior, and medial surfaces along with the ratio of these values (*in vivo* load/*in vivo* locomotion).

Bone surface	Froude class 5 (µε)	–100 N (µε)	Ratio
Anterior	645	403	0.62
Posterior	–448	–506	1.13
Medial	206	–52	–0.25

Values represent mean strain, and the ratio value was calculated as –100 N strain (µε) divided by Froude Class 5 strain (µε).

#### FEA validation during peak load

This study aimed to assess the correlation between experimentally measured and computationally modeled strain values at all gauge sites at the tibial midshaft. The objective was to better determine the distribution of strain across different surfaces of the tibia during peak loading conditions to enhance our understanding of the biomechanical behavior of the iguana tibia.

**Fig. 6:**
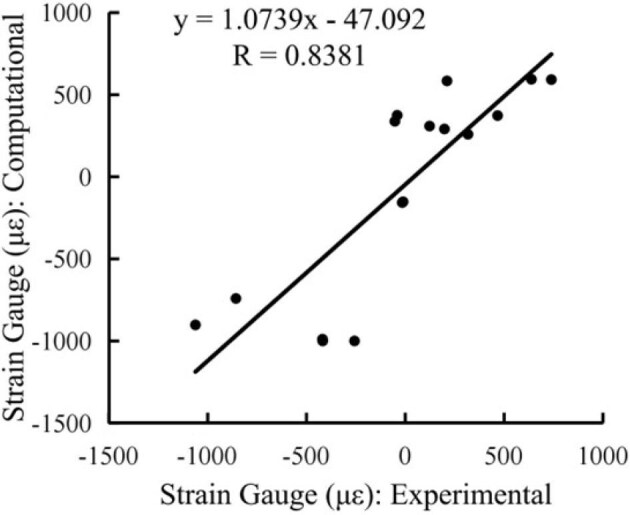
Scatterplot demonstrates the relationship between computationally modeled strain and experimentally measured strain. A trendline and equation with Spearman correlation coefficient (*R*) values are displayed. Data points represent surfaces from *n* = 6.

**Fig. 7:**
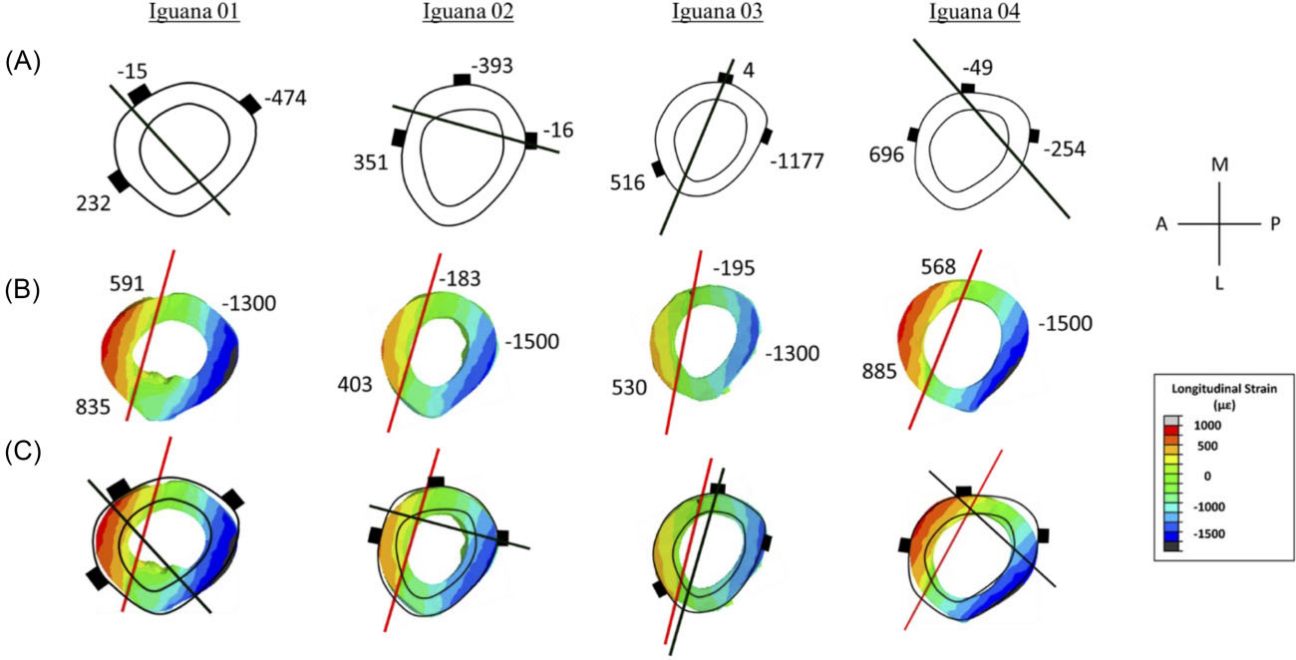
(**A**) Experimental neutral axis among individual iguanas during *in vivo* –100 N axial compressive loading at midshaft (50% of bone length from the proximal end). Results display all individual iguanas that had strain gauges on all surfaces (anterior, posterior, and medial) during peak load. Small boxes on the cortical surface represent the location represent locations of each strain gauge, and values represent the mean peak longitudinal strain (µε) for their respective strain gauges; positive values represent surfaces in tension, and negative values represent surfaces in compression. The neutral axis is represented by the solid black line, and the limb orientation is indicated by the legend. (**B**) Computational neutral axis among individual iguanas during –100 N axial compressive loading during finite element modeling at midshaft (50% of bone length from the proximal end). Results display all individual iguanas that had strain gauges on all surfaces (anterior, posterior, and medial) during peak load. Values represent mean longitudinal strain (µε) for its respective strain gauge; positive values represent surfaces in tension, and negative values represent surfaces in compression. The whole cortical bone strain (µε) at the midshaft volume of interest (VOI) is represented by colors; red/orange/yellow indicates tension, light blue/blue/black indicates compression, and green represents values near zero, as displayed on the scale. The neutral axis is represented by the solid red line, and the limb orientation is indicated by the legend. Finite element analysis (FEA) calculations are derived from 2.5% of the bone length (∼1.5 mm). (**C**) Overlaid comparison of experimental and computational tibial midshaft neutral axis among individual iguanas during –100 N axial compressive loading at midshaft (50% of bone length from the proximal end). The experimental neutral axis is represented by the solid black line, and the computational neutral axis is represented by the solid red line. Small boxes on each cortical surface indicate the location of strain gauges; orientation of limb represented by legend. The whole cortical bone strain (µε) at the midshaft VOI is represented by colors; red/orange/yellow indicates tension, light blue/blue/black indicates compression, and green represents values near zero, as displayed on the scale. FEA calculations are derived from 2.5% of the bone length (∼1.5 mm).

To validate the FE model of the green iguana tibial midshaft, results were compared between experimentally measured peak *in vivo* longitudinal strain and computationally modeled peak longitudinal strain by FE analysis. The experimentally measured strain values correlated closely with the computationally modeled strain values at the gauge sites, following linear regression (*y* = 1.0739*x* –47.092) and Spearman correlation (*R* = 0.8381) analysis (Fig. 6). Following validation, maximum and minimum peak principal strains were calculated at the cortical midshaft. Maximum principal strains were 426 ± 67 µε and minimum principal strains were –700 ± 96 µε (Table 5). For the four iguanas in which all three gauges were bonded during peak experimental loading, the strain distribution in the tibial midshaft showed that tensile strains were observed on the anterior surface, compressive strains on the posterior surface, and both tension and compression strains on the medial surface, indicating anterior-posterior directional bending (Fig. 7, Table 6). The medial surface was near the neutral axis, as observed during *in vivo* loading.

**Fig. 8:**
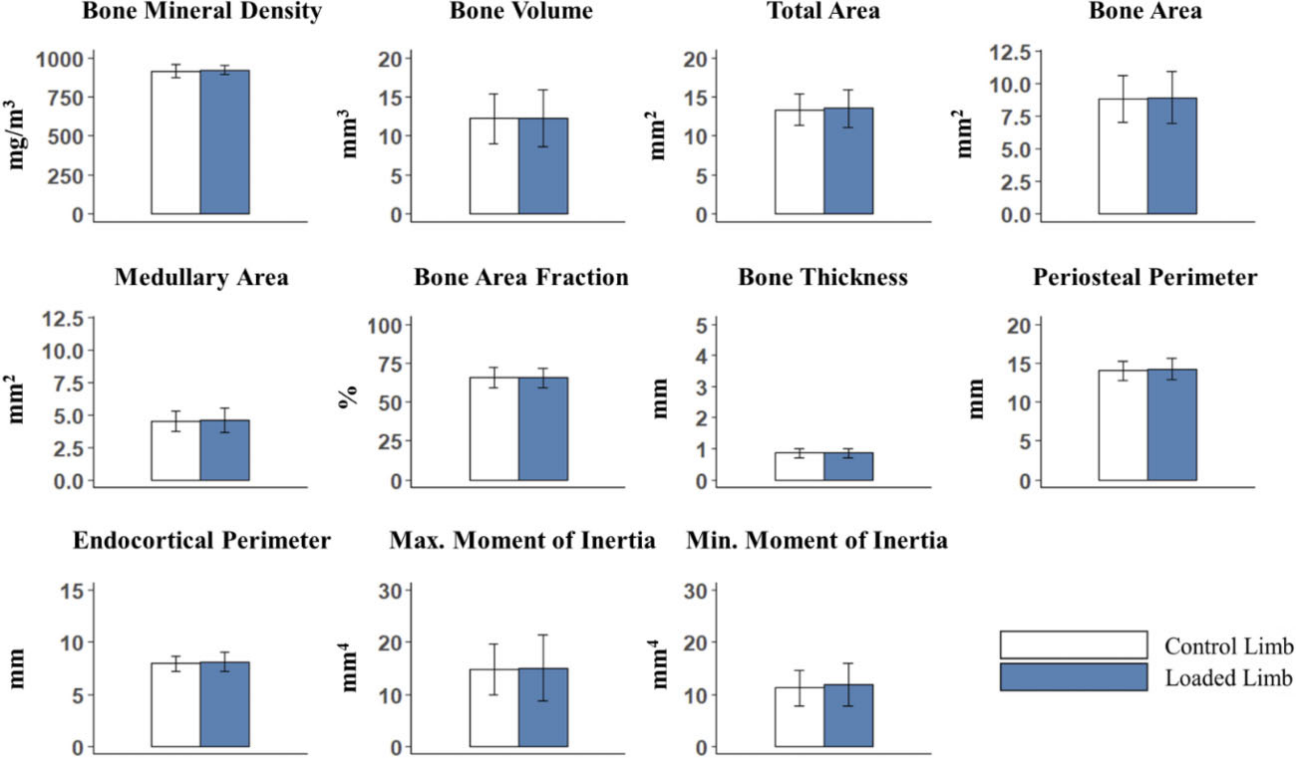
Cortical bone morphometry results by microcomputed tomography (µCT) for the green iguana tibia from 2.5% of the bone length, centered at 50% of bone relative to the proximal end (*n* = 9). Parameters included bone mineral density, bone volume, total area, bone area, medullary area, bone area fraction, bone thickness, periosteal perimeter, endocortical perimeter, and maximum and minimum moments of inertia. Blue bars represent the left, loaded hindlimbs, while the white bars represent the right, nonloaded limb. Statistical significance was determined by the application of a paired, two-tailed Student’s *t*-test or a Wilcoxon signed-rank test for each parameter between the loaded and control limbs (*P* < 0.05). No significance was detected by the *t*-test or the Wilcoxon sign-rank test.

**Table 5:** FE modeled average and 95th percentile maximum and minimum principal strains, along with their respective ± standard deviations at the tibial midshaft (50% of bone length). Calculations are derived from 2.5% of the bone length (∼1.5 mm).

Peak strains (µε) at the cortical midshaft		
	Maximum principal strain	Minimum principal strain
95th percentile	692 ± 162	–1633 ± 234
Mean	426 ± 67	–700 ± 96

**Table 6:** Peak FE analysis longitudinal strain for the green iguanas that had all three strain gauges bonded during peak *in vivo* compressive loading at the tibial midshaft (50% of bone length). Calculations are taken from 2.5% of the bone length (∼1.5 mm).

Iguana ID	Anterior (µε)	Posterior (µε)	Medial (µε)
1	835	–1300	591
2	403	–1500	–183
3	530	–1300	530
4	885	–1500	568

**Table 7:** Cortical bone morphometry results by microcomputed tomography (µCT) for the green iguana tibia from 2.5% of the bone length, centered at 50% of bone relative to the proximal end (*n* = 9) for both the left, loaded hindlimbs and the right, nonloaded control limbs. Parameters included bone mineral density, bone volume, total area, bone area, medullary area, bone area fraction, bone thickness, periosteal perimeter, endocortical perimeter, and maximum and minimum moments of inertia.

	Loaded limb	Control limb	*P*-value
Bone mineral density (mg/cm^3^)	921.12 ± 29.95	916.0 ± 43.54	0.65
Bone volume (mm^3^)	12.29 ± 3.63	12.21 ± 3.15	0.8
Total area (mm^2^)	13.53 ± 2.41	13.37 ± 1.99	0.56
Bone area (mm^2^)	8.94 ± 2.00	8.84 ± 1.79	0.6
Medullary area (mm^2^)	4.58 ± 0.93	4.53 ± 0.81	0.55
Bone area fraction (%)	65.74 ± 6.18	65.70 ± 6.44	0.91
Bone thickness (mm)	0.87 ± 0.15	0.86 ± 0.15	0.7
Periosteal perimeter (mm)	14.23 ± 1.39	14.03 ± 1.22	0.28
Endocortical perimeter (mm)	8.11 ± 0.96	7.98 ± 0.73	0.25
Maximum moment of inertia (mm^4^)	15.08 ± 6.41	14.86 ± 4.92	0.82
Minimum moment of inertia (mm^4^)	11.85 ± 4.15	11.22 ± 3.38	0.23

Values represent mean ± standard deviation. *P*-value results represent the application of a paired, two-tailed Student’s *t*-test and a Wilcoxon signed-rank, if necessary, for each parameter between the loaded and control limbs.

#### Tibial midshaft distribution at peak compressive loading: *in vivo* and FEA

The orientation of the neutral axis at midshaft during peak –100 N loading varied significantly between individuals during experimentally applied axial compressive loading (Fig. 7A). Under the same boundary conditions, the neutral axis calculated by the FE model did not differ at midshaft (Fig. 7B). Comparing the neutral axis at midshaft for both experimentally measured strains and FE model predicted strains, only one iguana had no difference (Fig. 7C).

## Cortical bone morphology—results

### Skeletal response to mechanical load

#### Effects of daily dynamic loading on cortical bone morphometry

With the objective of investigating the skeletal response to mechanical load, we focused next on bone morphometry analyses at localized bone sites. For the green iguana, daily applied dynamic loading did not have any significant morphological effect on the cortical bone VOI at either 37% or 50% (Table 7, Fig. 8) of the tibia’s length from its proximal end. Similar negative results from the 37% cortical bone VOI are reported in supplementary materials (Table S3, Fig. S1). Together these data suggest that the effectiveness of the axial tibial compressive loading model to induce a bone adaptive response may vary between vertebrate groups.

## Discussion

### Iguana locomotion

There have been few studies characterizing the *in vivo* tibial strain distribution of reptiles during locomotion. While the focus of our study was different from most locomotor studies, our results provide further insight into the ongoing effort to study reptile biomechanics during critical activities. During treadmill locomotion, the *in vivo* strain distribution exhibited distinct patterns across all bone surfaces even though strain distribution did not change with increasing speeds as the iguanas changed from a sprawling limb posture during slow walking to a more upright posture while sprinting at higher Froude classes. Our data adds to the previous nonmammalian (green iguana and American alligator) locomotor studies (Blob and Biewener 1999) that indicate strain magnitude does not decrease during an upright limb posture relative to sprawling locomotion as observed in mammalian species (Biewener 1989), adding to the biomechanical divergence seen between mammalian and reptilian species.

Tensile strain was observed on the anterior surface, compression on the posterior surface, and mainly tension with minimal compression on the medial surface. The strain distribution measured in our study aligns with a previous *in vivo* strain locomotor study of the iguana tibia, with both studies observing the tibia being loaded with net tension in bending in the anterior-medial to posterior-lateral direction (Blob and Biewener 1999). Additionally, both studies observed that peak longitudinal strains occur near mid-stance, displaying the greatest point of bending, presumably occurring when most of the weight of the hindlimb is firmly on the ground. In the same study (Blob and Biewener 1999), *in vivo* strain was measured at two speeds, with the aim of characterizing the strain during different limb postures during walking and running steps while we measured *in vivo* strains over varying speeds from a walk to a sprint. In the previous study, a considerable increase in tensile strain was observed on the anterior and medial surfaces and an increase in compression on the posterior surface. In contrast, our mean *in vivo* strain did not increase with increasing speeds on any surface.

During the running trials of this study, we observed longitudinal strain magnitudes near those reported by Blob and Biewener (+1650 µε) in two of our iguanas. We observed tensile strains during running to be +1444 µε and +1771 µε. Contrarily, we observed either compression strain or tensile strain in one iguana on the anterior surface, indicating on average, there may be a distinct locomotor loading pattern but that there is more variability in the cross-sectional strain distribution than expected. The average size of the iguanas in our study was larger, and as a group we observed lower mean longitudinal strain, and the higher strain magnitudes measured were from individual iguanas at the top of our size range, which may indicate that animal size plays an important role during locomotion.

Although there were no statistically significant differences in tibial strain across the five Froude classes, we observed a noticeable dip in longitudinal strain at Froude Class 3 on the anterior surface (Fig. 4A). It is difficult to make a direct comparison, as there are no other studies reporting hindlimb strain during this speed in green iguanas or other reptilian species, and this suggests the transition from sprawling to more upright may not be necessarily gradual as we assumed, indicating further kinematic studies are needed to assess hindlimb angle, ground reaction forces, and the interplay of muscles and bone during locomotion, allowing for comparison to previous studies (Blob and Biewener 2001). We were unable to bond rosette gauges to the anterior or posterior surfaces of the tibia due to surface availability; this limitation prevented us from assessing changes to the strain direction in more detail and may indicate that peak tensile or compression strain is not longitudinal. Torsional rotation may be influencing the transition, as torsion on the medial surface was highest during this speed (Table 2).

This trend may correspond to a gait transition zone, where the animal modifies its kinematic strategy, resulting in changes in limb posture, muscle recruitment, and load distribution—factors that could collectively reduce tibial strain. Schöner et al. (1990) proposed a synergetic theory of quadrupedal gait transitions, suggesting that animals pass through transitional speed zones in which the current gait becomes less stable while a new gait pattern begins to emerge. Such overlap may represent a dynamic instability that prompts a shift, for example, from a trot to a gallop. Froude Class 3 may therefore reflect a biomechanical transition point in iguana locomotion. We measured peak principal tensile and compression strains on the medial surface of the tibia, which is a novel finding in the field of reptile biomechanics. Our measured mean angle of E1 (ϕ1) did not indicate torsional rotation of the tibia, but we observed variation with some individual iguanas closer to 45° than the average, which is another indication that there is increased variability among iguanas. E1 angle decreased as speed increased but occurred relatively at similar times during stance (%), the tibia may rotate further due to the limb being in contact with the ground for a long period of time at the slower speeds compared to higher speeds. This relationship may be conclusively determined by future kinematic studies.

The high degree of variability in locomotor strains observed in our study may also contribute to the claim that biological structures select for higher safety factors, the ratio between the applied strain that results in fracture and daily physiological strains, when responding to variability in loads (Alexander 1981; Lowell 1985; Blob and Biewener 1999). Higher safety factors may be a protective measure to avoid injury during locomotion, but this was beyond the scope of this study.

Limitations of measuring *in vivo* strains using strain gauges include the need for extremely invasive surgery, which is only feasible when the bone surface permits secure attachment. This constraint prevented us from using all rosette strain gauges, which are larger and more difficult to implant, in all positions. We also had to use single-element gauges, acknowledging the potential limitation that their alignment may not have been perfectly parallel to the longitudinal axis of the bone. Moreover, it is technically too difficult to simultaneously apply a strain gauge to the fibula without disrupting the bonded gauges on the tibia or vice versa. We do not have *in vivo* strain data for the fibula, but as the fibula is posterior-lateral to the tibia, we can infer that the fibula may be loaded in compression during locomotion (Blob and Biewener 1999); however, additional *in vivo* results would be required to confirm.

Additionally, the variability observed and the small sample sizes in our *in vivo* study (*n* = 7) and in Blob and Biewener (*n* = 2–3) signal that further research is needed to conclusively determine specific trends in reptile hindlimb biomechanics during locomotion.

#### Bone strains in response to mechanical load

There are no previously published studies measuring the *in vivo* strain distribution in the iguana tibia during applied axial compressive loading. Therefore, our study presents a novel aspect of reptile bone biomechanics. The iguana tibia is loaded in bending during both locomotion and applied mechanical loading, but there is a change in bending direction comparing *in vivo* longitudinal strain during locomotion and applied compressive mechanical loading. During locomotion the bending direction is anterior-medial to posterior-lateral, while it is anterior-lateral to posterior-medial bending during applied loading. The cross-sectional neutral axis was not reported during locomotion, but this change indicates the tibia during axial compressive loading is subjected to nonphysiological conditions. Interestingly, the mean longitudinal *in vivo* strain magnitude linearly increases with increasing force, revealing a predictable relationship, differs from our locomotion results, assuming increasing speeds results in higher forces placed on the tibia during locomotion. Our results may indicate future studies will need to determine the role limb posture plays in applied force during varying locomotor speeds. As the limb appears to be loaded nonphysiologically, we would expect to detect higher longitudinal strains on all surfaces of the tibia, but that was not observed in our results. On the anterior surface, the mean longitudinal *in vivo* strain was lower in magnitude than the *in vivo* strain observed during running trials (Table 6), indicating that the fibula plays a more critical role in bearing compressive loads than we expected. As we do not have *in vivo* strain data of the fibula, the cross-sectional strain distribution suggests that the fibula may also be loaded in axial compression as inferred during locomotion.

It has been hypothesized that the curvature of long bones is developed to adapt to dynamic loading (Bertram and Biewener 1988), and applying bending moments in the mouse tibia has been observed to result in changes in cross-sectional geometry and strain magnitudes of the tibial cortical midshaft during growth (Main et al. 2010; Razi et al. 2015). The low strain magnitudes during axial compressive loading indicate that the fibula may prevent the tibia from being subjected to increased bending, as observed in the murine fused tibia and fibula, indicating key differences between natural and applied loading.

The anatomy of the knee joint may also play a critical role in the measured strain, as the customized knee cup of the loading fixtures contacted the distal femur, and the femur contacted the tibia toward the caudal end of the proximal tibia, not the centroid of the tibia. This may have resulted in the applied axial compressive loads not creating enough of a bending moment and inducing low strains. Assessing the influence of hindlimb muscle forces during applied axial compressive loading was beyond the scope of this study and may warrant investigation.

#### Computational and experimental bone strains

FE models are continually providing detailed characterizations of bone mechanobiology, and our results add to the continuing validation of previous FEA techniques. To date, no attempt has been made to computationally model the iguana tibia. µCT-based FEA combined with *in vivo* strain provides detailed strain patterns in localized VOIs in response to applied mechanical loading (Yang et al. 2014). Validating FEA results is critical for interpreting *in vivo* data and for analyzing a bone’s adaptive response to mechanical loading. As a novel model, it is critical that our FE model at the cortical midshaft of the green iguana tibia was validated, enhancing the accuracy of future models for the entire bone. To better reflect concordance between experimental and computational strain values, we report the Spearman correlation coefficient (*R* = 0.8381). While we retained Model I regression for consistency with prior biomechanical studies, Model II regression (e.g., reduced major axis) would be more appropriate for assessing agreement between datasets and should be considered in future work.

At the tibial midshaft, the FE model confirmed patterns with the *in vivo* measured strain during experimentally applied loading as the tibia was loaded in bending with the medial strain gauge near the neutral axis. The *in vivo* strains were lower than the measured strain present in our FE model, and the midshaft distribution showed a difference between the neutral axis of the experimentally and computationally measured strain from the same iguanas. Previous studies (Patel et al. 2014; Yang et al. 2014) have also shown that there is generally a high variation in measured strains with strain gauges during *in vivo* tibial loading experiments. Therefore, this difference is generally accepted. While the FE model measured strains were validated at the tibial midshaft, these results indicate some interesting differences. µCT scans included the fibula, which was included in modeling, but the reference point #1 at the proximal tibial articular surface and the direction of the applied load indicate that the applied load is transmitted through the tibia alone (Fig. 3), while the *in vivo* measured strain during experimentally applied loading indicates the fibula bears more mechanical load than assumed. As reported (Yang et al. 2014), the influence of soft tissues, ligaments, and cartilage was assumed to be negligible but may be more critical than previously suspected in reptiles compared to mammals. The musculature between individual iguanas also varied, with some hindlimbs being very thick and some thin, which may also have played a factor in reducing the induced strain on the tibia during experimentally applied loading compared to our computationally modeled strain.

At a peak load of –100 N, the tibial midshaft orientation of the neutral axis differed for three of the four iguanas that still had all three strain gauges bonded. One of the objectives of the mechanical loading fixtures and the purpose of applying a –10 N preload was to firmly secure the limb and the knee and ankle joints during loading. However, the iguana foot is quite wide due to the anatomy of their digits, which varied between individual iguanas. This variation may have played a factor in the exact contact point where the load was applied to the foot from the actuator. Further research may elucidate the impact of the design of the fixtures and the size range within the sample size.

Overall, the results from these studies indicate a correlation between experimentally measured *in vivo* longitudinal strains and computationally modeled strains during FEA of the green iguana tibia at the cortical midshaft. The strain distribution across different tibial surfaces during peak loading conditions revealed tensile strains observed on the anterior surface, compressive strains on the posterior surface, and a combination of tension and compression on the medial surface. This study adds specific knowledge on the biomechanical behavior of the green iguana tibia, validating the effectiveness of FEA in studying tibial strain dynamics.

#### Tissue-level skeletal response to mechanical load

Following 3 weeks of daily and dynamic loading, there was no significant difference detected between any cortical bone morphological parameters at the 37 and 50% VOI of the iguana tibia (Table 7); however, potential explanations were identified related to the green iguana as a representative species.

At the 37% VOI, decreased BMD, bone volume, bone area fraction, bone thickness, and maximum moment of inertia indicated that the green iguana tibia had adapted to respond to mechanical loading and resorption had occurred during remodeling, while newly deposited bone had not been mineralized or detected in response to the applied loading (Table S3, Fig. S1). The decrease in maximum moments of inertia, along with decreased bone volumes, indicated a reduced resistance to bending due to the decrease in volume, as there was no significant difference in the periosteal and endocortical perimeters. Due to difficulty in bonding a strain gauge to the 37% VOI due to the green iguana musculature, we were not able to measure *in vivo* strain in response to locomotion and axial compressive loading. Measuring the cortical morphology may better demonstrate that this site may experience higher strains at this location compared to the midshaft, as seen in murine models (Sugiyama et al. 2010) and is sensitive to applied axial compressive loading and could be the target site for future studies in reptiles, facilitating cross-species comparisons.

The anatomy of reptiles is different from other vertebrate groups, and this may have influenced how they responded to mechanical load. As previously discussed, the femur contacts the caudal surface of the proximal tibia, and the midshaft of the iguana tibia is generally anatomically straight and may be less susceptible to bending moments compared to the more proximal VOI and may account for the lack of significant differences observed in all cortical morphological parameters. As we observed no significant difference in all cortical bone morphological parameters at midshaft, the results may indicate difficulties regarding utilizing the axial compressive tibial loading model with the green iguana as a representative species.

Our study attempted to apply the optimal mechanical loading protocol; however, our results may indicate that the axial compressive tibial loading model may not be an effective mechanical loading model to transmit the strain magnitudes necessary to induce a bone adaptive response in green iguanas. Despite our best efforts to quantify the applied load magnitude and number of cycles per day, the low *in vivo* strains measured during experimentally applied compressive loading compared to the *in vivo* strains measured during treadmill locomotion, and the lack of a cortical bone response indicates that the green iguana hindlimb may be more adapted to bear compressive mechanical loads compared to other vertebrate groups.

A limitation of assessing the tissue-level response through µCT scans is the resolution of the scans. For our FE model, 60 µm resolution was chosen to allow us to limit the number of scans to stitch together to three per iguana, which was a resolution large enough to subreconstruct and scan the hindlimb joints. To retain the same resolution throughout the study, 60 µm was used for bone morphology analysis. A previous study assessing bone formation in iguanas observed that cortical bone formation rates varied from 0.4 to 3.4 µm/day (Green et al. 2023). This may indicate that our scan resolution over the 3-week study period, with a total of 22 days from Day 1 of loading to euthanasia, might not have been adequate to observe a bone adaptive response in cortical bone of the green iguana tibia. The length of daily applied loading of 3 weeks was selected from previous axial tibial compressive loading in other nonmammalian species (Verner 2017) and may need to be increased for future studies. Other limitations include not assessing the cancellous bone response in either the distal or proximal epiphyses, as our aim of the study focused on the cortical bone morphological response in common tibial strain-gauged VOIs that would allow for comparison across various models.

## Conclusion

This study is an early attempt to characterize the *in vivo* strain of the iguana tibia across increasing speeds during locomotion, axial compressive loading, and FEA and to apply the measured *in vivo* results to a multiweek daily, dynamic mechanical loading protocol to characterize the cortical bone response. The *in vivo* results during locomotion contribute to the limited existing research on the biomechanics of reptiles, while the mechanical loading data and FE model are novel and shed more light on the musculoskeletal mechanobiology of reptiles. Our studies have attempted to enhance understanding of reptilian skeletal adaptation and redirect efforts toward a model that can offer us a broader understanding of the physiological animal skeletal systems across taxa.

Despite the value of our study, there are limitations that should be acknowledged to emphasize the need to focus future efforts on investigating the evolutionary biomechanical and physiological mechanobiology dynamics of nonmammalian and nonavian models. Although we were unable to observe the cortical bone response hypothesized following the mechanical loading protocol, future studies could refine our protocol, potentially measuring the response using the noninvasive axial compressive tibial loading model to assess the bone adaptive response in both cortical and cancellous bone. There is a need to close the knowledge gap across all vertebrate groups when investigating skeletal health and biomechanics. Our *in vivo* strain data and FEA characterize the mechanical environment of the green iguana tibia and do not include the fibula, which our results have indicated may play a more critical role in bearing mechanical load in green iguana compared to mice or birds. Future studies investigating the *in vivo* strain of the green iguana hindlimb may need to collect *in vivo* strain of the green iguana fibula to potentially gather critical information regarding the relationship between the tibia and fibula in green iguana biomechanics and mechanobiology.

Our novel results represent significant progress in understanding biomechanical and mechanobiological mechanisms in reptiles and how these adaptive mechanisms meet the environmental demands placed upon the skeleton and provide insights into utilizing the reptile model to better understand bone evolutionary dynamics and skeletal health.

## Supplementary Material

obaf036_Supplemental_File

## Data Availability

The datasets used for this study are available from the corresponding author upon reasonable request.
