# Supplementary material for: Tibial Biomechanics and Adaptive Response to Mechanical Stimuli in the Green Iguana
Source: Integr Org Biol. 2025 Oct 22;7(1):obaf036. doi: 10.1093/iob/obaf036 (PMC12550686; doi:10.1093/iob/obaf036)
Supplement: obaf036_Supplemental_File [file obaf036_supplemental_file.docx]

**Supplementary Materials**

**Table S1.** Individual green iguana sizes (n=7) used during *in vivo* strain (µε) analysis following treadmill locomotion and experimentally applied axial compressive loading and used for FE modeling. SVL is snout-vent length and TL is tibial length. Mass is in kilograms, SVL is snout-vent length in millimeters and TL is tibial length in millimeters. Final values equal mean ± one standard deviation.

| **Iguana** | **Mass (kg)** | **SVL (mm)** | **TL (mm)** |
| --- | --- | --- | --- |
| 1 | 0.7 | 246 | 53 |
| 2 | 0.64 | 245 | 46 |
| 3 | 0.7 | 227 | 50 |
| 4 | 0.96 | 285 | 60 |
| 5 | 0.9 | 285 | 55 |
| 6 | 1.2 | 337 | 61 |
| 7 | 1.06 | 325 | 55 |
| Mean ± SD | 0.88 ± 0.19 | 278.57 ± 38.73 | 54.29 ± 4.89 |

**Table S2.** Individual green iguana sizes (n=9) used during cortical bone morphometry analysis following a three-week, daily axial compressive loading protocol. SVL is snout-vent length and TL is tibial length. Mass is in kilograms, SVL is snout-vent length in millimeters and TL is tibial length in millimeters. Final values equal mean ± one standard deviation.

| **Iguana** | **Mass (kg)** | **SVL (mm)** | **TL (mm)** |
| --- | --- | --- | --- |
| 8 | 0.94 | 290 | 56 |
| 9 | 0.78 | 265 | 55 |
| 10 | 1.1 | 300 | 57 |
| 11 | 1.1 | 312 | 60 |
| 12 | 1.02 | 280 | 56 |
| 13 | 0.72 | 247 | 53 |
| 14 | 1.18 | 306 | 61 |
| 15 | 0.7 | 255 | 50 |
| 16 | 1.22 | 295 | 62 |
| Mean ± SD | 0.97 ± 0.19 | 283.33 ± 21.77 | 56.67 ± 3.65 |

**S3**

In this study, the relationship between peak *in vivo* longitudinal midshaft tibial bone strains and increasing magnitudes of axial compressive mechanical loads was to be applied to determine the necessary load magnitude required to induce a bone response at functional strain levels similar to those applied in mammals and birds. The initial objective was to apply a mechanical load that would induce peak strains that were 2.5x greater than the *in vivo* longitudinal peak strains observed during locomotion; specifically, those peak strains observed in top speed trials (Froude Class #5). Following analysis of *in vivo* longitudinal strains during mechanical loading*,* it was observed that the highest applied mechanical load magnitude of -100N did not induce strains 2.5x greater than the *in vivo* longitudinal strains observed during locomotion on any bone surfaces.

To determine the required axial compressive mechanical loading protocol to induce a daily strain for an intracortical bone response, we used the daily strain stimulus approach ([Chen et al. 2010](#_Chen_JC,_Beaupré)). It was determined during *in vivo* loading of the first iguana group that a load of -75N could be applied daily without incurring damage to the soft tissue and bones of the iguana hindlimb. After analysis of the FEA model, peak *in vivo* longitudinal strains were observed to be the highest on the posterior surface. Determination of the number of daily cycles to be applied had two-fold components: (1) Applying the observed *in vivo* longitudinal strain on the posterior surface (-381 µε) during -75N axial compressive mechanical loading in iguanas to observed daily strain stimulus (number of cycles (n) * *in vivo* longitudinal surface strain (µε)). This approach was informed by previous axial compressive loading protocols and observed strain on the posterior surface during applied axial compressive mechanical loading in Chukars, which elicited a cortical bone adaptive response in the Chukar tibiotarsus (216 cycles, -526 µε) ([Verner 2016](#_Verner_KA._2016.)). Strain and cycles were given the same importance, so the strain-cycle weighting exponent (*m*) was given a value of 1.0.

(1) [(216 cycles) (-526 µε) = [(# of cycles) (-381 µε)]

-113,616 µε =-381 µε (# of cycles)

**n = 298 cycles/day**

(2) To estimate the required maximum daily strain stimulus, we used a cycle-strain relationship calculation, utilizing data from turtles and iguanas. We applied the observed peak *in vivo* longitudinal strain observed in reptiles such as turtles (+2374 µε) ([Butcher et al. 2008](#_Butcher_MT,_Espinoza)) and in iguanas (+1650 µε) ([Blob and Biewener 1999](#_Blob_RW,_Biewener_1)) during running steps. These strains were identified as the highest reported strains in iguanas. We then applied this stimulus to the 216 cycles used in previous axial compressive loading models in birds and mammals ([Verner 2016](#_Verner_KA._2016.)). Strain and cycles were given the same importance, so the strain-cycle weighting exponent (*m*) was given a value of 1.0.

(2) [(216) (+2374 µε) = [(# of cycles) (+1650 µε)]

+512,784 µε = +1650 µε (# of cycles)

**n = 311 cycles/day**

To determine if 300 daily cycles were safe to apply to iguanas, a daily loading protocol of 300 cycles at -75N was applied to iguana cadaver whole limbs (n=3) for 5 days and µCT scanned for any structural damage. Following scanning, no damage was observed.

The target size for this group of iguanas was based on the ratio of *in vivo* peak longitudinal strain during treadmill locomotion and *in vivo* peak longitudinal strain during experimentally applied mechanical loading. To calculate the ratio the mean peak longitudinal strain observed at -75N and -100N load magnitudes was divided by the mean peak longitudinal strain observed during the top speed (Froude Class #5). After analysis of the calculated ratio for individual iguanas, the individual iguana (Mass: 0.7 kg, SVL: 246.0 mm, TL: 53.0 mm) who had the highest values during -75N and -100N loading on the anterior (1.3 and 1.83) and posterior (1.54 and 2.08) surfaces was selected as the target size for the iguanas in our cortical bone morphology study following an applied mechanical loading protocol.

# **Table S3** Cortical bone morphometry results by microcomputed tomography (µCT) for the green iguana tibia from 2.5% of the bone length, centered at 37% of bone relative to the proximal end (n=9) for both the left, loaded hindlimbs and the right, non-loaded control limbs. Parameters included bone mineral density, bone volume, total area, bone area, medullary area, bone area fraction, bone thickness, periosteal perimeter, endocortical perimeter, and maximum and minimum moments of inertia. Values represent mean ± standard deviation. P-value results represent the application of a paired, two-tailed Student’s t-test for each parameter between the loaded and control limbs.

|  | **Loaded Limb** | **Control Limb** | **P-Value** |
| --- | --- | --- | --- |
| **Bone Mineral Density (mg/cm^3^)** | 931.72 ± 29.19 | 941.76 ± 29.09 | 0.07 |
| **Bone Volume (mm^3^)** | 13.23 ± 3.42 | 13.36 ± 3.41 | 0.053 |
| **Total Area (mm^2^)** | 14.82 ± 2.55 | 14.86 ± 2.49 | 0.76 |
| **Bone Area (mm^2^)** | 9.65 ± 1.82 | 9.89 ± 1.9 | 0.11 |
| **Medullary Area (mm^2^)** | 5.14 ± 1.07 | 4.96 ± 0.89 | 0.16 |
| **Bone Area Fraction (%)** | 65.01 ± 4.27 | 66.33 ± 4.62 | 0.08 |
| **Bone Thickness (mm)** | 0.89 ± 0.12 | 0.92 ± 0.13 | 0.09 |
| **Periosteal Perimeter (mm)** | 15.01 ± 1.43 | 14.97 ± 1.37 | 0.84 |
| **Endocortical Perimeter (mm)** | 8.63 ± 0.89 | 8.51 ± 0.79 | 0.17 |
| **Maximum Moment of Inertia (mm^4^)** | 17.94 ± 5.79 | 19.12 ± 6.28 | 0.056 |
| **Minimum Moment of Inertia (mm^4^)** | 14.25 ± 5.32 | 13.38 ± 4.85 | 0.36 |

**
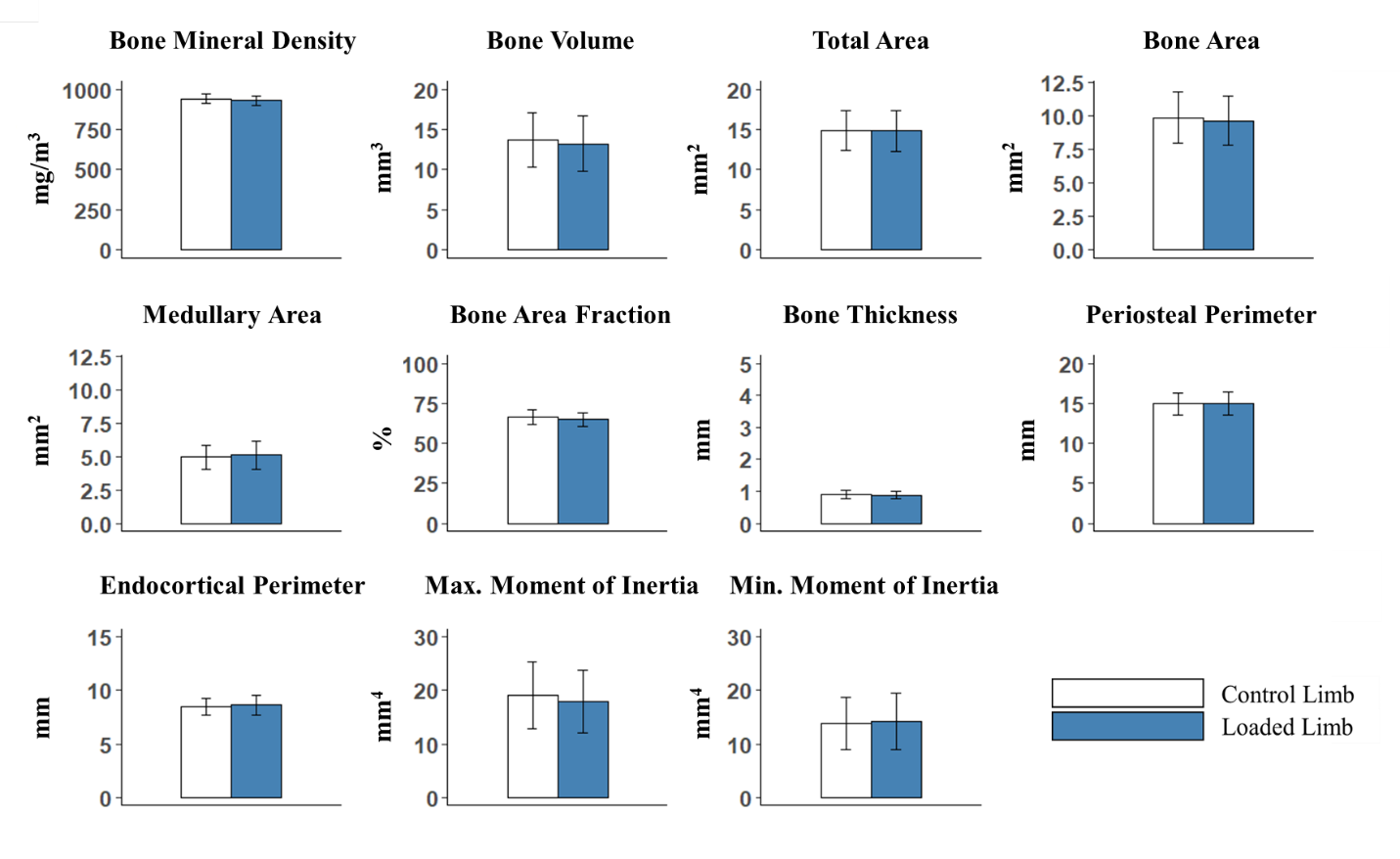
**

**mg/cm^3^**

# **Figure S1** Cortical bone morphometry results by microcomputed tomography (µCT) for the green iguana tibia from 2.5% of the bone length, centered at 37% of bone relative to the proximal end (n=9). Parameters included bone mineral density, bone volume, total area, bone area, medullary area, bone area fraction, bone thickness, periosteal perimeter, endocortical perimeter, and maximum and minimum moments of inertia. Blue bars represent the left, loaded hindlimbs, while the white bars represent the right, non-loaded limb. Statistical significance was determined by the application of a paired, two-tailed Student’s t-test for each parameter between the loaded and control limbs (p<0.05). No significance was detected by the t-test.
